# Supplementary material for: The gut microbiota and depressive symptoms across ethnic groups
Source: Nat Commun. 2022 Dec 6;13:7129. doi: 10.1038/s41467-022-34504-1 (PMC9726934; doi:10.1038/s41467-022-34504-1)

Supplementary Information: The gut microbiota and depressive symptom levels across ethnic groups

J.A. Bosch et al.

Supplementary Table 1. Results of linear regression models comparing results for Shannon's and Simpson's index with depressive symptom scores as dependent variable or predictor, respectively.

**Model**	**unadjusted**		**Model 1**		**Model 2**		**Model 3**	
	Beta	*p-value*	*Beta*	*p-value*	*Beta*	*p-value*	*Beta*	*p-value*
**Alpha-diversity as predictor of depression scores**								
Shannon	-.120	<.001	-.073	<.001	-.060	.002	-.042	.026
Simpson	-.097	<.001	-.055	.002	-.046	.012	-.037	.038
**Depression scores as a predictor of alpha diversity**								
Shannon	-.120	<.001	-.066	<.001	-.054	.002	-.039	.026
Simpson	-.097	<.001	-.055	.002	-.045	.012	-.038	.038

Supplementary Figure 1.

Figure 1 panel A and B present the association (rho) between depression symptom score (GHQ-9) and the Shannon index stratified by ethnicity. Heterogeneity statistics are presented in boxes. Panel **A** provides unadjusted regression coefficients and panel **B** provides age, gender, and education-adjusted coefficients (Model 1) with confidence intervals. Dot indicates r (effect size), dot size corresponds with sample size, whiskers indicate 95% CI. **Panel C** provides a stratified overview of median values (IQR in box) of Shannon index, whiskers indicate 1.5 x IQR whereby dots are exceeding that value. N = 769 for Dutch, 527 South-Asian Surinamese, 767 African Surinamese, 458 Ghanian, 349 Turkish and 473 Moroccan.

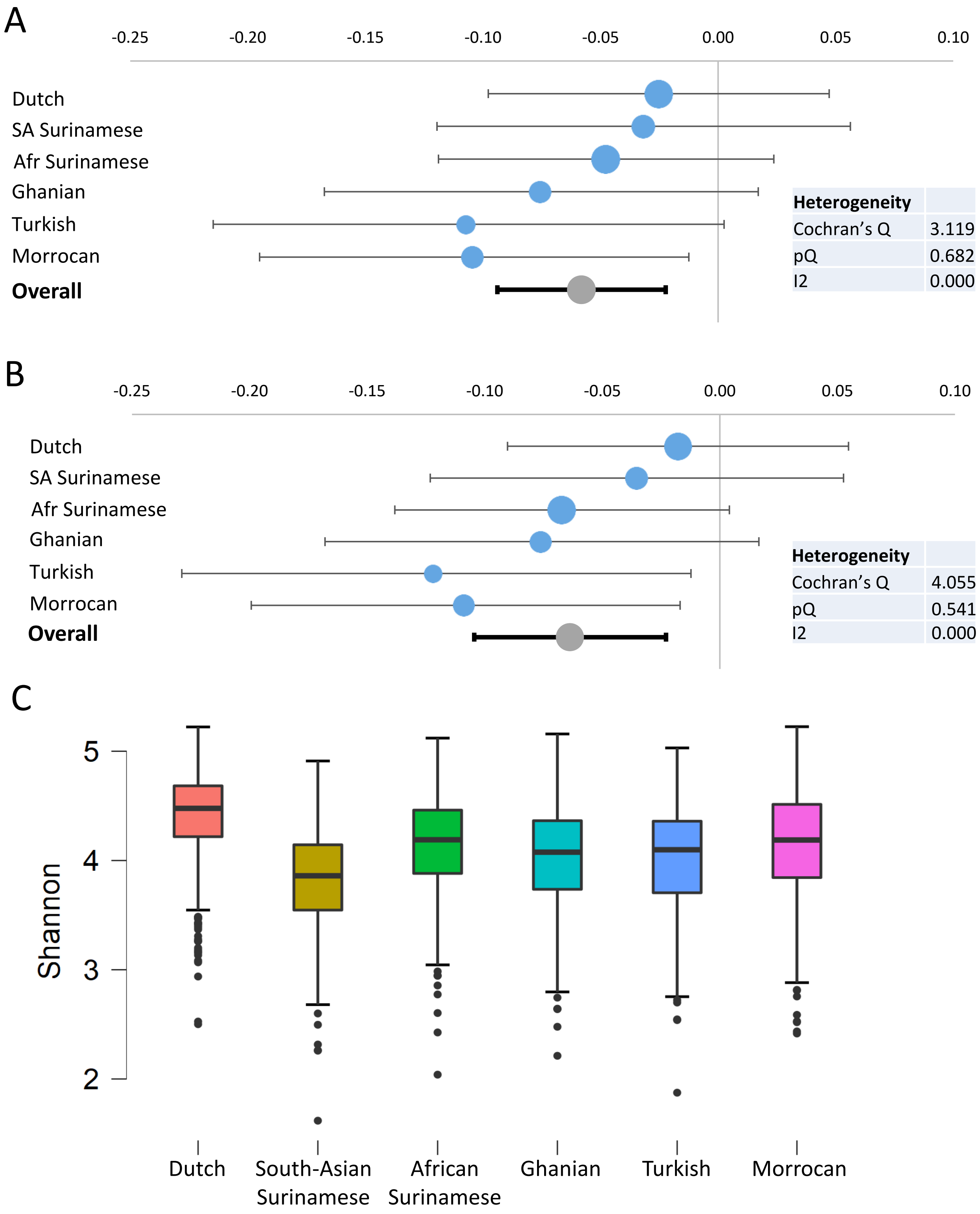

Supplementary Figure 2.

Figure 2 presents scatter plots depicting associations (Rho) of individual taxa with depression (PHQ-9) and alpha-diversity (Shannon). Each dot represents a taxonomic unit (ASV). Axes show the size of the correlation between each ASV and the indicated parameter. Plots depict only ASVs that; 1) could be identified to least a Genus level and; 2) whereby at least one ASV within that genus was significantly associated with depression scores. Species names are added when available.

Panel A presents the associations for genera of the phylum Firmicutes (see Supplemental Tables) whereas **Panel B** presents the associations for the other phyla. The Shannon index is presented on the y axis. As shown in **Panel C**, the Shannon index highly correlated with Bray-Curtis Beta-diversity PC2 (also see the corresponding results section).

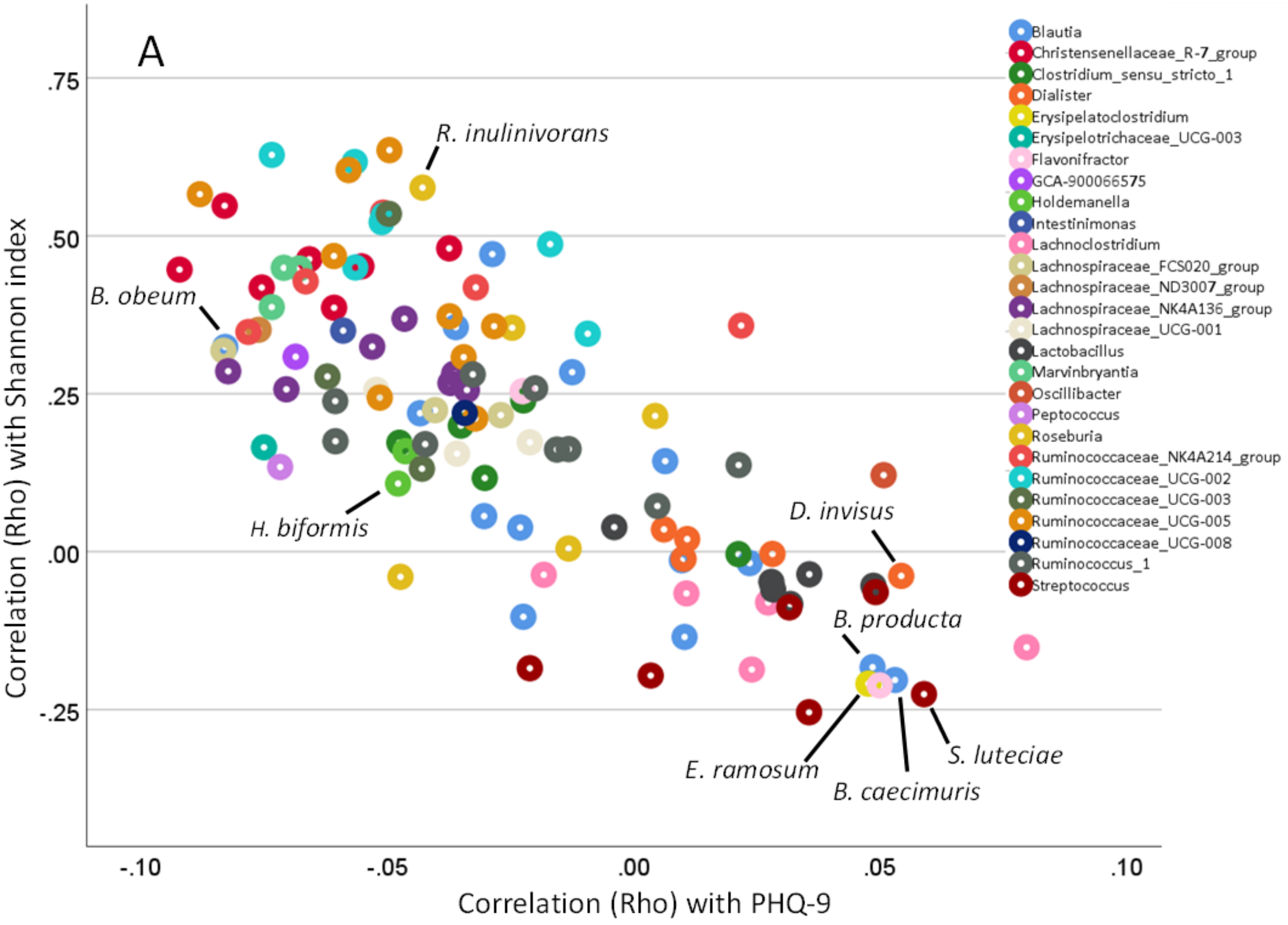

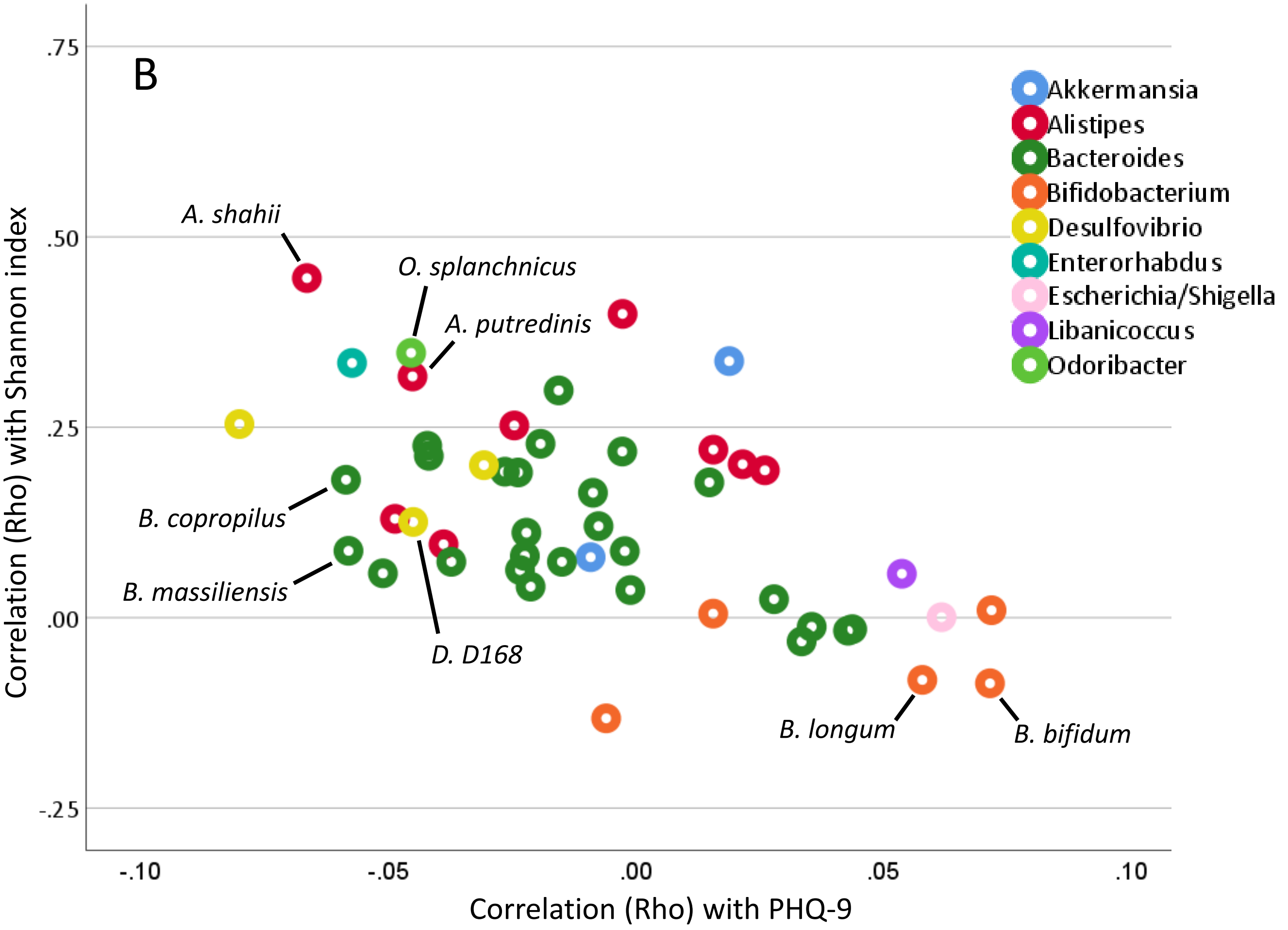
B
Akkermansia
Alistipes
Bacteroides
Bifidobacterium
Desulfovibrio
Enterorhabdus
Escherichia/Shigella
Libanicoccus
Odoribacter
A. shahii
O. splanchnicus
A. putredinis
B. copropilus
B. massiliensis
D. D168
B. longum
B. bifidum
.75
.50
.25
.00
-.25
-.10
-.05
.00
.05
.10
Correlation (Rho) with Shannon index
Correlation (Rho) with PHQ-9

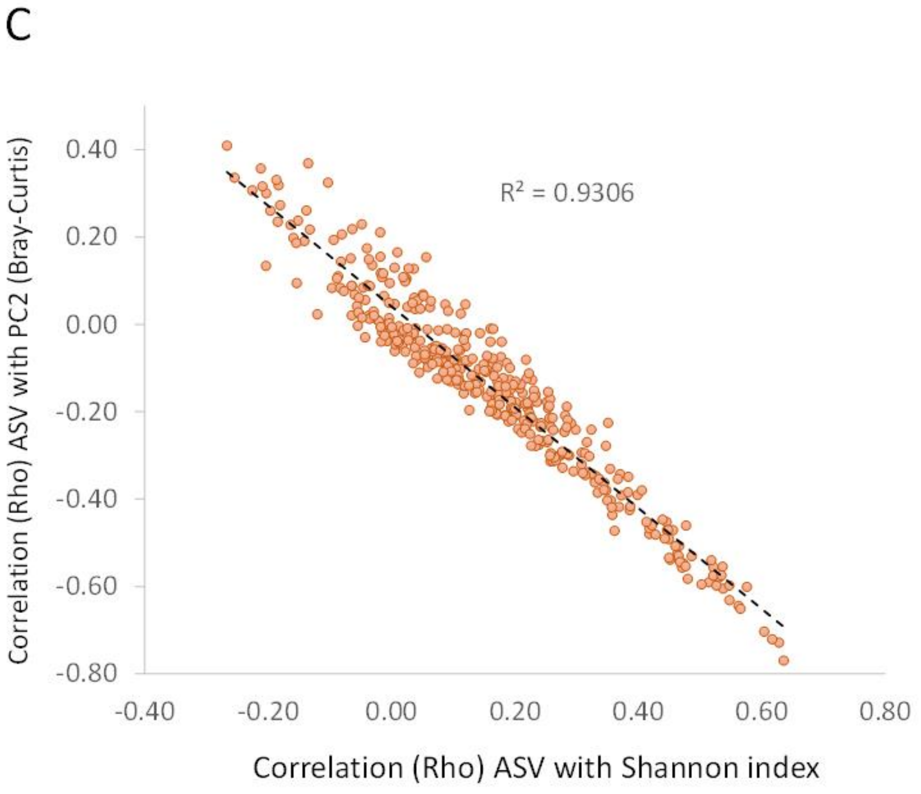
C
R² = 0.9306
0.40
0.20
0.00
-0.20
-0.40
-0.60
-0.80
-0.40
-0.20
0.00
0.20
0.40
0.60
0.80
Correlation (Rho) ASV with PC2 (Bray-Curtis)
Correlation (Rho) ASV with Shannon index

Supplementary Figure 3. For illustrative purposes, this figure presents scatter plots showing corresponding associations of alpha diversity with depressive symptom scores and a selected depression risk factor. The overall picture emerges that ASVs associated with alpha diversity also tend to exhibit a corresponding association with common depression risk factors. A more complete overview is provided in the supplementary tables. Each dot represents a taxonomic unit (ASV). The axes scale the strength of the association (Rho) between each ASV and the indicated parameter.

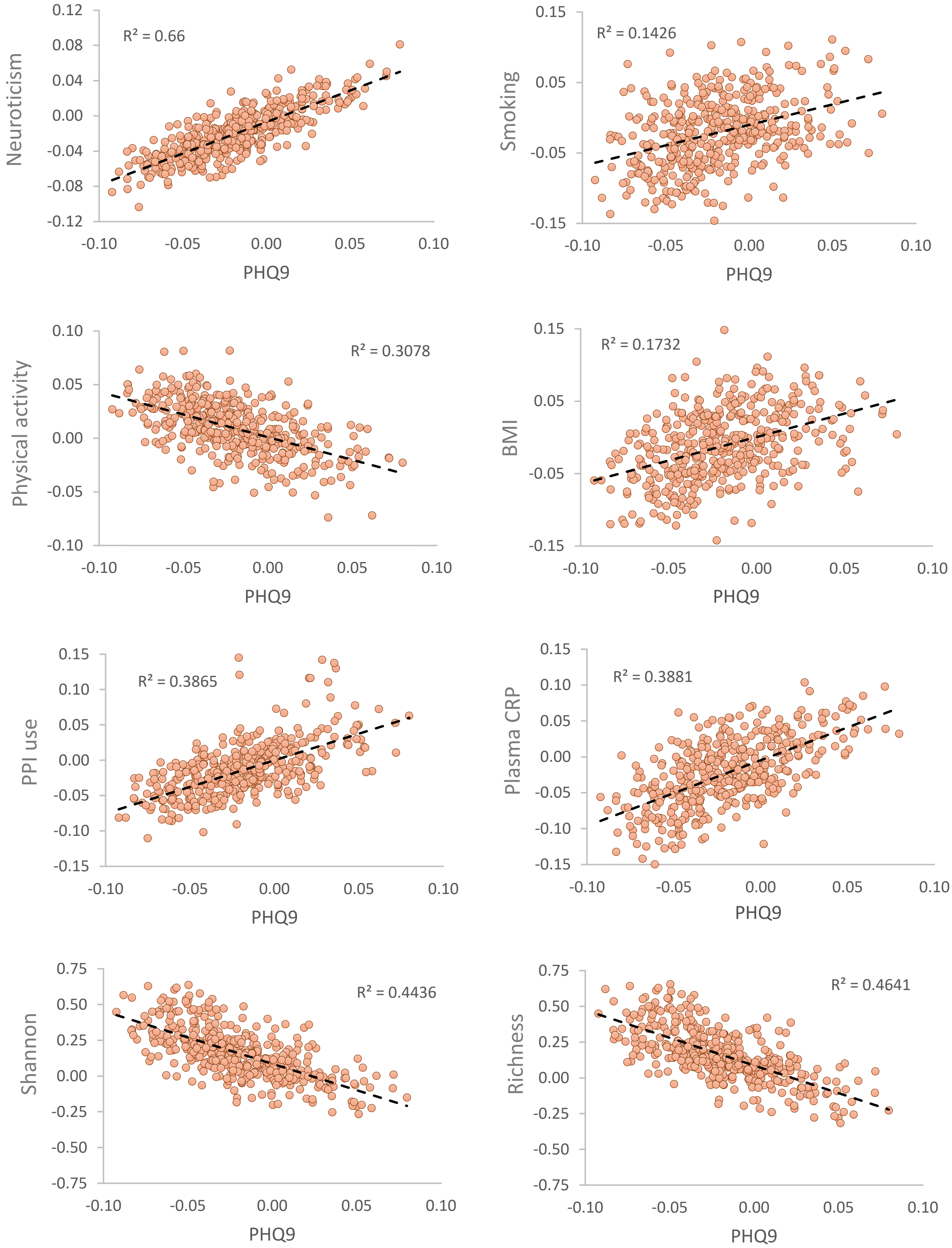

Supplement: Supplementary file 1 — Supplementary Information [file 41467_2022_34504_MOESM1_ESM.pdf]
