# Supplementary material for: The gut microbiota and depressive symptoms across ethnic groups
Source: Nat Commun. 2022 Dec 6;13:7129. doi: 10.1038/s41467-022-34504-1 (PMC9726934; doi:10.1038/s41467-022-34504-1)
Supplement: Supplementary file 3 — Description of Additional Supplementary Data Files [file 41467_2022_34504_MOESM3_ESM.docx]

**Description of Additional Supplementary File (*.xls)**

Tab name: Supplementary Data file 1

Description: Source data of Figure 1

Tab name: Supplementary Data file 2

Description: Source data of Figure 2. This data file is a subset of data presented in “Supplementary Data 3”.

File name: Supplementary Data file 3

Description: Source file of the supplementary Figures. A selection of this data is presented in Figure 2, and Supplementary Data files 4 and 5, which present relevant sections of the data in Table format (see legend with Supplementary Data file 4 and 5 for a full description)

File name: Supplementary Data 4

Description: This file provides a table view of source data file “Supplementary Data 3”; it presents an overview of associations of all ASV (rows) with PHQ-9 scores, covariates and relevant risk factors and relevant correlates (in columns). The table is organized (top to bottom) in order of strength of associations with PHQ9. This Data file also expands the data presented in Figure 2 of the manuscript, which only shows associations that were significant (FDR adjusted) applying regression model 0.

Regression analyses (Models 1 – 3) were applied only to ASVs that were significantly associated (FDR <.05) with depressive symptom levels in the unadjusted analyses (presented in column “unadjusted (model 0)”). “*Core*” highlights ASVs with >75% prevalence in the sample population (indicated by green check mark). “*Heterogeneity*” highlights ASV’s showing a moderate (yellow flag: I^2^ between 30% and 50%) and high level of ethnic heterogeneity (red flag: I^2^ >50%). Numbers in heat maps indicate Spearman’s Rho of each ASV with the indicated parameter. For interpretation of the direction of the association; with each parameter higher scores indicate higher values, and sex was scored 0 or 1 for women and men, respectively. For all other bivariate parameters 0 is “No” and 1 “Yes”. By approximation, a Rho β of |.043| reflects a statistically significant association (FDR adjusted per column).

File name: Supplementary Data 5

Description: : This file provides a table view of source data file “Supplementary Data 3”; it presents an overview of associations of all ASV (rows) with PHQ-9 scores, covariates and relevant risk factors or correlates (in columns). The table is ordered alphabetically according to taxonomic classification. This Data file also expands the data presented in Figure 2 of the manuscript, which only shows associations that were significant (FDR adjusted) applying regression model 0.

Regression analyses (Models 1 – 3) were applied only to ASVs that were significantly associated (FDR <.05) with depressive symptom levels in the unadjusted analyses (presented in column “unadjusted (model 0)”). “*Core*” highlights ASVs with >75% prevalence in the sample population (indicated by green check mark). “*Heterogeneity*” highlights ASV’s showing a moderate (yellow flag: I^2^ between 30% and 50%) and high level of ethnic heterogeneity (red flag: I^2^ >50%). Numbers in heat maps indicate Spearman’s Rho of each ASV with the indicated parameter. For interpretation of the direction of the association; with each parameter higher scores indicate higher values, and sex was scored 0 or 1 for women and men, respectively. For all other bivariate parameters 0 is “No” and 1 “Yes”. By approximation, a Rho β of |.043| reflects a statistically significant association (FDR adjusted per column).
