# Supplementary material for: The gut microbiota and depressive symptoms across ethnic groups
Source: Nat Commun. 2022 Dec 6;13:7129. doi: 10.1038/s41467-022-34504-1 (PMC9726934; doi:10.1038/s41467-022-34504-1)
Supplement: Supplementary file 5 — Reporting summary [file 41467_2022_34504_MOESM5_ESM.pdf]

nature portfolio

Corresponding author(s): Jos A. Bosch

Last updated by author(s): Jul 14, 2022

Reporting Summary

Nature Portfolio wishes to improve the reproducibility of the work that we publish. This form provides structure for consistency and transparency in reporting. For further information on Nature Portfolio policies, see our Editorial Policies and the Editorial Policy Checklist.

Statistics

For all statistical analyses, confirm that the following items are present in the figure legend, table legend, main text, or Methods section.

n/a	Confirmed	
☐	☒	The exact sample size (*n*) for each experimental group/condition, given as a discrete number and unit of measurement
☐	☒	A statement on whether measurements were taken from distinct samples or whether the same sample was measured repeatedly
☐	☒	The statistical test(s) used AND whether they are one- or two-sided<br>*Only common tests should be described solely by name; describe more complex techniques in the Methods section.*
☐	☒	A description of all covariates tested
☐	☒	A description of any assumptions or corrections, such as tests of normality and adjustment for multiple comparisons
☐	☒	A full description of the statistical parameters including central tendency (e.g. means) or other basic estimates (e.g. regression coefficient) AND variation (e.g. standard deviation) or associated estimates of uncertainty (e.g. confidence intervals)
☐	☒	For null hypothesis testing, the test statistic (e.g. *F*, *t*, *r*) with confidence intervals, effect sizes, degrees of freedom and *P* value noted<br>*Give P values as exact values whenever suitable.*
☒	☐	For Bayesian analysis, information on the choice of priors and Markov chain Monte Carlo settings
☒	☐	For hierarchical and complex designs, identification of the appropriate level for tests and full reporting of outcomes
☐	☒	Estimates of effect sizes (e.g. Cohen's *d*, Pearson's *r*), indicating how they were calculated

Our web collection on statistics for biologists contains articles on many of the points above.

Software and code

Policy information about availability of computer code

Data collection	No software has been used for data collection
Data analysis	A description of software and algorythms is presented in the methods section of the manusctipt.<br>Raw sequencing reads were quality checked using FastQC. USEARCH (v11.0.667 64-bit Linux version) was used to process the raw reads. UNOISE3 algorithm (α-parameter set to 2.0) was used to infer biological sequences (α-parameter set to 2.0). Taxonomy was assigned to the ASVs with the SINTAX algorithm using Greengenes v.13.5 and Silva 132. Rarified ASV sequences were then used as input for MAFFT (v.7.427) in order to obtain a multiple sequence alignment, based on which a phylogenetic tree was constructed using IQ-TREE (v. 1.6.11) . The phylogenetic tree was midpoint-rooted using the “phytools” R package. The “phyloseq” R package was used to integrate the ASV counts, taxonomy assignments, phylogenetic tree and sample metadata.<br>Statistical analyses and data visualisations were performed in R version 4.0.1, SPSS v27, or JASP 0.13.1. All information and code is made availalble through an online repository Software description and syntax of analyses can be found at https://amcmc.github.io/HELIUS_depression/

For manuscripts utilizing custom algorithms or software that are central to the research but not yet described in published literature, software must be made available to editors and reviewers. We strongly encourage code deposition in a community repository (e.g. GitHub). See the Nature Portfolio guidelines for submitting code & software for further information.

Data

Policy information about availability of data

All manuscripts must include a data availability statement. This statement should provide the following information, where applicable:

- Accession codes, unique identifiers, or web links for publicly available datasets
- A description of any restrictions on data availability
- For clinical datasets or third party data, please ensure that the statement adheres to our policy

The HELIUS study has an open policy with regard to collaboration with other research groups and welcomes collaborations from a wide variety of disciplines. Information on procedures to acquire the available data and/or samples are described in the HELIUS Collaboration Policy, which can be found at www.heliusstudy.nl/nl/researchers/collaboration. In brief, to make use of the available data for research, we request a publication proposal describing background, aim, research questions, methods (analysis plan), and time table. All proposals should be submitted to HELIUS via the corresponding author of this article, or via info@heliusstudy.nl. The proposals are discussed in the HELIUS Executive Board regarding the study aims (compatibility with the general objectives of the HELIUS study/informed consent), the quality of the research proposal, and potential overlap with on going studies. After approval, the requested data will be provided after a Data Transfer Agreement has been signed. For more information, please visit our website or contact the corresponding author.

Human research participants

Policy information about studies involving human research participants and Sex and Gender in Research.

Reporting on sex and gender	Sex/gender was determind by self report. Analyses adjusted for potential sex biases by including sex in the main analyses. The relevant regression weights are reported in in Table 2a and Table 2b. Supplemental Tables and accompanying Source Data file (excel) also separately report correlations (Rho) between sex and microbial parameters.
Population characteristics	Participants aged 18–70 years were first stratified by ethnic origin, and subsequently randomly sampled within each stratum, sthrough the municipal registry of Amsterdam. Included were Amsterdam residents of Dutch, Surinamese, Ghanaian, Turkish and Moroccan ethnic origin. A full description of the cohort and sampling strategy is provided in: Snijder, M. B. et al. Cohort profile: the Healthy Life in an Urban Setting (HELIUS) study in Amsterdam, The Netherlands. BMJ Open 7, e017873, doi:10.1136/bmjopen-2017-017873 (2017). The current analytical subsample involved those for which fecal samples were collected. At the time of the present analyses, fecal 16S rRNA data were available for a total of 3.343 participants belonging to 8 ethnic groups. Because of small numbers, those identifying as Indonesian-Surinamese background (N= 46) and "another or unknown ethnicity" (N= 63) were excluded. Applying these criteria, and excluding those without data on depressive symptoms (N=93), yielded the following 6 ethnic groups; Dutch (N=769), African Surinamese (N=767), South-Asian Surinamese (N=527), Turkish (N=349), Moroccan (N=473), and Ghanaian (N=458). A detailed description of the analytical sample and covariates included in the analyses is provided in Table 1 of the manuscript.
Recruitment	See above. Detailed information on participantion rate (N=24,789) and biases in response rate (28%) is provided in Snijder, M. B. et al. Cohort profile: the Healthy Life in an Urban Setting (HELIUS) study in Amsterdam, The Netherlands. BMJ Open 7, e017873, doi:10.1136/bmjopen-2017-017873 (2017).
Ethics oversight	The Medical Ethics Review Board of the Amsterdam University Medical Center https://www.amc.nl/web/research-75/medisch-ethische-toetsings-commissie-metc.htm

Note that full information on the approval of the study protocol must also be provided in the manuscript.

Field-specific reporting

Please select the one below that is the best fit for your research. If you are not sure, read the appropriate sections before making your selection.

☐ Life sciences ☒ Behavioural & social sciences ☐ Ecological, evolutionary & environmental sciences

For a reference copy of the document with all sections, see nature.com/documents/nr-reporting-summary-flat.pdf

Behavioural & social sciences study design

All studies must disclose on these points even when the disclosure is negative.

Study description	Crossectional population cohort analysis
Research sample	The HELIUS (Healthy Life in an Urban Setting) study is a multi-ethnic cohort study among citizens of Amsterdam, The Netherlands. Participants aged 18–70 years were first stratified by ethnic origin, and subsequently randomly sampled within each stratum, sthrough the municipal registry of Amsterdam. Included were Amsterdam residents of Dutch, Surinamese, Ghanaian, Turkish and Moroccan ethnic origin. A full description of the cohort and sampling strategy is provided in: Snijder, M. B. et al. Cohort profile: the Healthy Life in an Urban Setting (HELIUS) study in Amsterdam, The Netherlands. BMJ Open 7, e017873, doi:10.1136/bmjopen-2017-017873 (2017). The current analytical subsample involved those for which fecal samples were collected. At the time

	of the present analyses, fecal 16S rRNA data were available for a total of 3.343 participants belonging to 8 ethnic groups. Because of small numbers, those identifying as Indonesian-Surinamese background (N= 46) and "another or unknown ethnicity" (N= 63) were excluded. Applying these criteria, and excluding those without data on depressive symptoms (N=93), yielded the following 6 ethnic groups; Dutch (N=769), African Surinamese (N=767), South-Asian Surinamese (N=527), Turkish (N=349), Moroccan (N=473), and Ghanaian (N=458). A detailed description of the analytical sample is provided in Table 1 of the manuscript.
Sampling strategy	See above and Snijder, M. B. et al. Cohort profile: the Healthy Life in an Urban Setting (HELIUS) study in Amsterdam, The Netherlands. Participants aged 18–70 years were randomly sampled, stratified by ethnic origin, through the municipal registry of Amsterdam. BMJ Open 7, e017873, doi:10.1136/bmjopen-2017-017873 (2017).
Data collection	Data were collected by interview, questionnaire, physical examination, and from biological analyses of serum/plasma and fecal samples. Interviewers were of the same ethnic backround as the interviewees. See also: Snijder, M. B. et al. Cohort profile: the Healthy Life in an Urban Setting (HELIUS) study in Amsterdam, The Netherlands. BMJ Open 7, e017873, doi:10.1136/bmjopen-2017-017873 (2017).
Timing	Data were collected between January 2011 and December 2013
Data exclusions	Because of small numbers, those identifying as Indonesian-Surinamese background (N= 46) and "another or unknown ethnicity" (N= 63) were excluded from the present analyses. Also excluded were those without data on depressive symptoms (N=93)
Non-participation	Detailed information on participantion rate (N=24,789) and response rate (28%) is provided in Snijder, M. B. et al. Cohort profile: the Healthy Life in an Urban Setting (HELIUS) study in Amsterdam, The Netherlands. BMJ Open 7, e017873, doi:10.1136/bmjopen-2017-017873 (2017).
Randomization	N/A: this is a population cohort study, there are no conditions over which to randomize

Reporting for specific materials, systems and methods

We require information from authors about some types of materials, experimental systems and methods used in many studies. Here, indicate whether each material, system or method listed is relevant to your study. If you are not sure if a list item applies to your research, read the appropriate section before selecting a response.

Materials & experimental systems

n/a	Involved in the study
☒	☐ Antibodies
☒	☐ Eukaryotic cell lines
☒	☐ Palaeontology and archaeology
☒	☐ Animals and other organisms
☒	☐ Clinical data
☒	☐ Dual use research of concern

Methods

n/a	Involved in the study
☒	☐ ChIP-seq
☒	☐ Flow cytometry
☒	☐ MRI-based neuroimaging
